# Pepsinogen A, pepsinogen C, and gastrin as markers of atrophic chronic gastritis in European dyspeptics

**DOI:** 10.1038/sj.bjc.6600877

**Published:** 2003-04-15

**Authors:** N Broutet, M Plebani, C Sakarovitch, P Sipponen, F Mégraud, and the Eurohepygast Study Group

**Affiliations:** 1 Unité d'Epidémiologie des Maladies Digestives, Laboratoire de Bactériologie, Université Victor Segalen Bordeaux 2, 146, rue Léo Saignat, 33076 Bordeaux, Cedex, France; 2 Department of Laboratory Medicine, University Hospital of Padova, Italy; 3 Jorvi Hospital, Espoo, Finland

**Keywords:** *Helicobacter pylori*, atrophic gastritis, pepsinogen, diagnosis, biomarkers

## Abstract

Serum levels of pepsinogen and gastrin are parameters that can be used as biomarkers for gastric mucosa. The aim of this study was to validate these serum biomarkers, that is pepsinogen A (PGA), pepsinogen C (PGC), PGA/PGC ratio, and gastrin, as screening tests for precancerous lesions: atrophic chronic gastritis (ACG) or *Helicobacter pylori*-related corpus-predominant or multifocal atrophy. The study population was comprised of a subsample of 284 patients from the 451 included in the Eurohepygast cohort, between 1995 and 1997. The concentrations of PGA, PGC, and gastrin were measured by radioimmunoassays. Histological diagnosis was the gold standard. Cut-off points were calculated using receiving operator characteristics (ROC) curves. Factors linked to variation of biomarkers were identified using multivariate linear regression. The mean of each biomarker in the sample was: PGA, 77.4 *μ*g l^−1^; PGC, 13.2 *μ*g l^−1^; PGA/PGC, 6.7; and gastrin, 62.4 ng l^−1^. For ACG patients, the areas under the PGA, PGC, PGA/PGC, and gastrin ROC curves were 0.55, 0.62, 0.73, and 0.58, respectively. The best cut-off point for PGA/PGC was 5.6, with sensitivity 65% and specificity 77.9%. For *H. pylori*-related corpus-predominant or multifocal atrophy, the areas under the respective ROC curves were 0.57, 0.67, 0.84, and 0.69. The best cut-off point for PGA/PGC was 4.7, with sensitivity 77.1% and specificity 87.4%. The results suggested that only the PGA/PGC ratio can be considered as a biomarker for precancerous lesions of the stomach, and may be useful as a screening test.

A number of studies pointed out the importance of atrophic chronic gastritis (ACG) in the evolution of chronic gastritis towards intestinal-type gastric adenocarcinoma (Correa *et al*, 1975,1990a).

The prevalence of ACG in the general population is still difficult to evaluate, since most of the data currently available were obtained from endoscopic studies of patients with digestive complaints. Only a few major studies have been conducted in the Finnish (Ihamäki *et al*, 1978,^1985^; Kekki *et al*, 1987) and in the Columbian (Correa *et al*, 1990b) populations. The difficulty in performing follow-up studies that would provide insight into risk factors, natural history, or the impact of *Helicobacter pylori* eradication on ACG arises from a lack of noninvasive tests, owing to the need of endoscopies to obtain biopsies for histological diagnosis.

Pepsinogen A (PGA) is secreted only by the chief cells of the corpus, and its serum level decreases with increasing grade of the corpus atrophy (loss of oxyntic glands) (Asaka *et al*, 1992). It is also an indirect measure of the corpus mass. Pepsinogen C (PGC) is secreted by antral glands, corpus chief cells, and the duodenal bulb in large quantities, and therefore PGC is not a direct measure of corpus atrophy (Plebani, 1993). Given that the majority of previous studies were performed on patients with pernicious anaemia or their relatives (Samloff *et al*, 1982; Borch *et al*, 1989), or individuals from populations with a high risk of gastric adenocarcinoma (Miki *et al*, 1987), that is subjects with severe corpus atrophy, PGA was a more interesting marker than PGC. Furthermore, the first studies that validated biomarkers used serological techniques and histological classifications, which have changed. In the present study, neither PGA nor PGC alone were good markers, contrary to the PGA/PGC ratio. Indeed, *H. pylori* infection normally progresses from the antrum to the corpus and, consequently, atrophy is more common in the antrum or both the antrum and the corpus than in the corpus alone (Sipponen *et al*, 1991).

In addition to epidemiological need, there are three other main reasons underlying the necessity of a practical, reliable, and inexpensive noninvasive screening test: (a) the association between ACG and intestinal-type gastric adenocarcinoma; (b) the prognosis of surgical treatment of gastric adenocarcinoma, which depends on the precocity of the detection of lesions (Craanen *et al*, 1991); and (c) the screening for ACG in patients from high gastric risk groups for gastric cancer, after eradication of *H. pylori* (El-Omar *et al*, 2000). The enzymes PGA and PGC and the hormone gastrin are good candidates for such a test, and have been evaluated in several countries. The characteristics of the tests varied widely between studies, and consistently did not identify good strategies for screening ACG (Borch *et al*, 1989; Kekki *et al*, 1991; Palli *et al*, 1991; Miki *et al*, 1995; Knight *et al*, 1996; Oksanen *et al*, 2000; Ley *et al*, 2001).

Eurohepygast is a large European multicentre cohort, whose aim is to study the natural history of chronic gastritis, and for whom a large database is available (Eurohepygast Study Group, 2002). Using these data at patients' inclusion, the aim of the present analysis was to evaluate the performance of the biomarkers PGA, PGC, PGA/PGC, and gastrin in discriminating between nonatrophic chronic gastritis (non-ACG) and ACG in this European dyspeptic population who have consulted gastroenterologists.

## PATIENTS AND METHODS

### Sample

**Figure 1:**
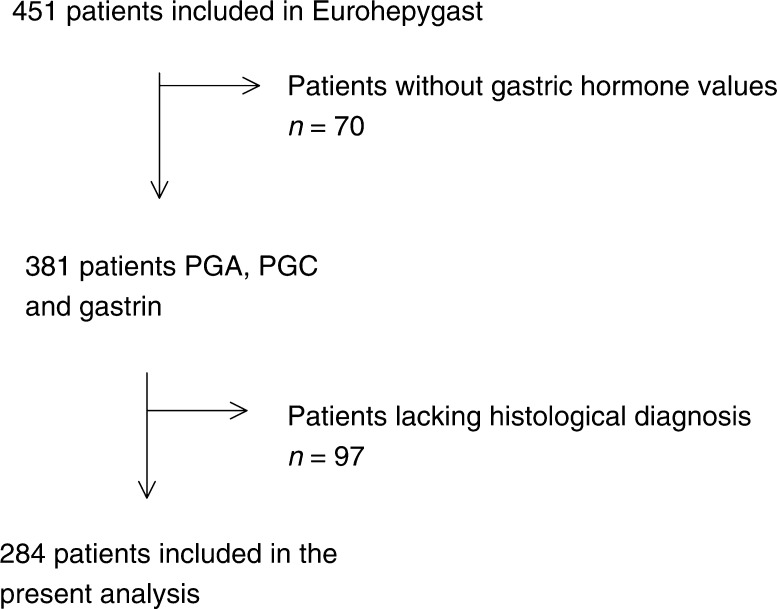
Patients included in the Eurohepygast study and reasons for exclusion of the present study.

This cross-sectional study was based on data from the Eurohepygast cohort collected between 1995 and 1997 at the inclusion of patients. A total of 451 patients were enrolled from 14 different European countries. To be included, patients with dyspepsia had to present themselves at one of the 19 participating European centres, to be between 18 and 75 years of age, and to have received no previous *H. pylori* eradication treatment nor proton pump inhibitors. Endoscopy with biopsies was performed. A questionnaire was filled out by the clinician and a blood sample was taken from nonfasting patients. Only a subsample of 284 patients was included in this study (Figure 1

).

### Endoscopy

Four biopsies were taken from the mid-antrum, two for histology and two for culture (Mégraud *et al*, 1999). Four biopsies were taken from the greater curvature of the mid-corpus, two for histology and two for culture. Biopsies for histology were placed in 10% formalin, and the remaining were immediately frozen at −80°C. Macroscopic findings were recorded according to the Sydney classification system for endoscopy (Tytgat, 1991).

### Histopathological diagnosis

For histological diagnoses, biopsies were routinely embedded in paraffin blocks, then sectioned and stained in each local centre. The stained slides were subsequently sent to a central laboratory, and examined by a single gastrointestinal pathologist (PS). All histopathological characteristics and parameters from the antrum as well as the corpus were graded and classified as normal mucosa, non-ACG, or ACG according to the initial and updated Sydney System (Price, 1991; Dixon *et al*, 1996). Normal mucosa or non-ACG was defined by the absence of any form of antral atrophy and no lesion in the corpus. ACG was defined as any form of moderate or severe atrophy with or without intestinal metaplasia and associated with chronic inflammation. *H. pylori*-related corpus-predominant atrophy was classified in ACG as well as multifocal ACG.

*H. pylori*-related corpus-predominant or corpus-limited atrophic gastritis is an existing topographic type of chronic gastritis (Correa, 1989). In this type, the morphology of the antral and corpus biopsies resembles that of the autoimmune atrophic gastritis: atrophy of advanced grade is seen predominantly or only in the corpus biopsies. Instead, or in the coassociation with autoimmune phenomena, there is evidence (usually increased antibody titres) of an ongoing *H. pylori* infection in these patients.

### Sera

Serum was kept at 4°C for a maximum of 24 h until transfer to a −80°C freezer. The serum samples were then transferred to the coordinating centre in dry ice, and stored at –80°C. At a later date, they were sent again in dry ice to a central facility in Italy for determination of the concentrations of the various parameters. The mean duration of storage at –80°C was 1 year and 9 months, and ranged from 7 months to 2 years and 9 months.

### Measurement of gastric enzymes and hormone

After thawing, sera were assayed blindly. PGA, PGC, and gastrin were measured by radioimmunoassays (Sorin Biomedica, Saluggia, Italy) (Plebani *et al*, 1996). The interassay coefficient of variation (CV) ranged from 7.4 to 9.5% for PGA levels between 20 and 120 *μ*g l^−1^, from 5.2 to 8.3% for PGC levels between 2.0 and 50 *μ*g l^−1^, and from 5.5 to 9.5% for gastrin levels between 40 and 250 ng l^−1^.

### Serological testing for *H. pylori* and CagA

Anti-*H. pylori* antibodies were detected by enzyme immunoassays (EIA): Pyloriset EIA-G test (Orion Diagnostica, Espoo, Finland) (Granberg *et al*, 1993); and by immunoblotting using Chiron RIBA *H. pylori* SIA (Chiron Diagnostics, Emeryville, CA, USA) (Yamaoka *et al*, 1998). Anti-CagA antibodies were detected by immunoblotting using Chiron RIBA *H. pylori* SIA, an in-house immunoblotting method performed in one of the centralised facilities (Figura *et al*, 1998), and by a modified in-house enzyme-linked immunosorbant assay (ELISA) (Heikkinen *et al*, 1997).

To be classified positive for *H. pylori* infection, concordant results of the Pyloriset EIA-G test and the Chiron RIBA *H. pylori* SIA were required. If they were in disagreement, the result of serology was considered to be undetermined. A similar approach was applied to the CagA status based on the result of the three tests. The first two tests performed were the Pyloriset EIA-G test and the in-house anti-CagA antibodies ELISA and, in case of disagreement, the Chiron RIBA *H. pylori* SIA was then carried out.

### Culture of *H. pylori*

Biopsies obtained from the antrum and the corpus were cultured locally according to a common protocol (Mégraud *et al*, 1999). Briefly, biopsies were ground with an electric tissue homogeniser (Ultraturax, LaboModerne, Paris, France) before inoculation onto a selective medium made of Wilkins Chalgren agar (Oxoid, Basingstoke, Hampshire, England, UK) enriched with 10% human blood and rendered selective by the addition of the antibiotics vancomycin (10 mg l^−1^), cefsulodin (5 mg l^−1^), trimethoprim (5 mg l^−1^), and cycloheximide (100 mg l^−1^). The plates were incubated under microaerobic conditions at 37°C for up to 12 days. The organisms were identified as *H. pylori* by Gram staining, and urease, oxidase, and catalase activity.

### Definition of *H. pylori* status

Patients were considered to be infected with *H. pylori* if one of the three tests was positive: culture, serology, or histology. Patients were considered to be noninfected when two of the three tests were negative, and the result of the third was either negative, undetermined, or missing.

### Data analysis

All statistical analyses were performed employing the STATA 5.0 software (Stata Corporation, College Station, TX, USA). Instead of considering only PGA and PGC alone, the PGA/PGC ratio was used. PGA and PGA/PGC had normal distributions but, since the distributions of PGC and gastrin were skewed on the right, a log transformation was used when the assumption of normality was needed. The means of these variables were compared between two groups by Student's *t*-tests (PGA and PGA/PGC ratio) or Mann–Whitney tests (PGC and gastrin); and comparisons between more than two groups were made with ANOVA (PGA and PGA/PGC ratio) or Kruskal–Wallis (PGC and gastrin) (Altman, 1991a). Correlation coefficients between two continuous variables were calculated as recommended by Altman (1991b).

The evaluation of the performance of gastric hormone concentrations as biomarkers for ACG was performed using histological diagnosis as the gold standard. The cut-off point for each of the biomarkers was determined by means of a receiving operating characteristic (ROC) curve. It was defined as the best point able to discriminate between the different histological patterns of patients with ACG and with normal mucosa or non-ACG (Altman, 1991c).

The area under the ROC curve measured the discriminatory ability of the model. General rules for the interpretation of the value of this area were applied (Hosmer and Lemeshow, 2000). For areas less than 0.7 the model did not discriminate; for areas 0.7, 0.8, and 0.9 the discrimination was acceptable, excellent, and outstanding, respectively. The cut-off value of each test was used to calculate the sensitivity, specificity, positive predictive value (PPV), negative predictive value (NPV), and accuracy of the test; 95% confidence intervals were calculated subsequently.

Different variables influencing the values of the biomarkers were identified with multivariate linear regressions adjusted by histological diagnosis. All variables with *P*<0.25 in univariate analyses were included in the complete multivariate linear model. Multivariate linear regressions were then performed using a backward elimination procedure (*P*>0.05). The performances of the biomarkers were then recalculated after adjustment by the remaining variables of the final linear model.

### Variables in the database

The 34 variables used in the univariate analyses were classified into five groups. The first group included all variables related to sociodemographic factors, namely, country of residence, country of birth, socioeconomical status, composition of family, profession, and level of education. The second group included variables related to behaviour: smoking habits (non-, ex-, or current smoker); consumption of alcohol (everyday, occasionally, or weekend only), sleeping tablets, and tranquillisers; and anxiety score (Covi *et al*, 1979). The third group corresponded to host-related variables, including body mass index (BMI), blood pressure, ABO and rhesus blood group, family history of digestive disease, and history of present disease. The fourth group comprised variables linked to diet. The fifth group was divided into three strata: *H. pylori*-negative patients, *H. pylori*-infected and CagA-negative patients, and *H. pylori*-infected and CagA-positive patients.

### Ethics

The study protocol was approved by the local Ethics Committee of each participating hospital. At inclusion into the study, each patient was required to sign an informed consent form.

## RESULTS

### Description of each biomarker

PGA, PGC, PGA/PGC, and gastrin values were available from 381 of the 451 patients included in the study. The mean age of these 381 patients was 43.5 years (s.d.=13.4 years), and ranged from 19 to 75 years. There were 138 (36.2%) men. In all, 115 (30.2%) of the patients were born in Western Europe and 249 (65.3%) in Eastern Europe. Concerning *H. pylori* status, 37 (10.0%) of the patients were not infected, 102 (27.7%) were infected but without anti-CagA antibodies, and 229 (62.2%) were infected and had anti-CagA antibodies. For 13 patients, the CagA status was unknown. The histology of 42 patients showed a normal mucosa, of 180 non-ACG, and of 62 ACG. Among the latter group, 28 patients had antral predominant atrophy, 27 *H. pylori*-related corpus-predominant atrophy, and seven multifocal ACG. In 97 patients, the histological diagnosis could not be performed properly.

**Figure 2:**
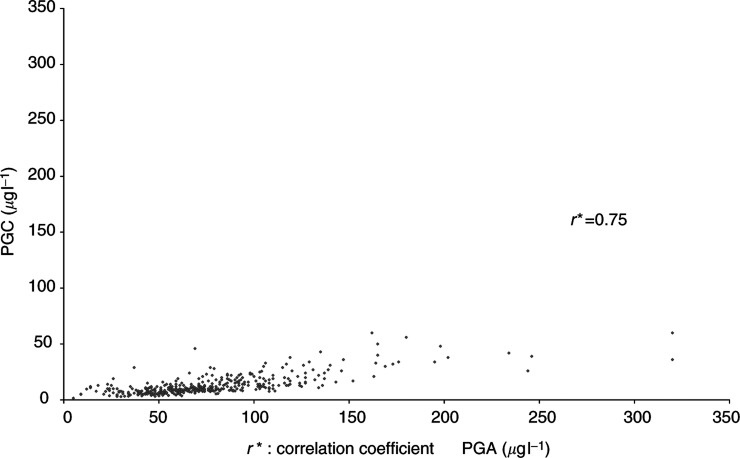
Plot of the serum values of pepsinogen (PG) A and PGC in 451 dyspeptic patients. Results of the Eurohepygast study. Coefficient correlation=0.75.

The mean (s.d.) of PGA, PGC, PGA/PGC, and gastrin in the sample (*n*=381) was 77.4 *μ*g l^−1^ (41.2), 13.2 *μ*g l^−1^ (9.0), 6.7 (2.6), and 62.4 ng l^−1^ (25.3), respectively. PGA and PGC were highly correlated, with a correlation coefficient of 0.75 (Figure 2

). Neither PGA nor gastrin was correlated to age (correlation coefficient: 0.05 and 0.10, respectively), but PGC increased with age (correlation coefficient: 0.23) and PGA/PGC decreased with age (correlation coefficient: –0.24).

The mean values for each of the biomarkers, PGA, PGC, PGA/PGC, and gastrin, differed significatively according to *H. pylori* and CagA status of the patients (*P*=0.02, 0.001, <0.0001, and 0.001, respectively) (Table 1

**Table 1:** Mean and standard deviation (s.d.) of pepsinogen (PG) A, PGC, and PGA/PGC ratio in the global sample and according to histological diagnosis in European dyspeptic patients. Results of the Eurohepygast study

	***n***	**PGA (*μ*g l^−1^) (s.d.)^a^**	**PGC (*μ*g l^−1^) (s.d.)^a^**	**PGA/PGC (s.d.)**^a^	**Gastrin (ng l^-1^) (s.d.)**^a^
Global sample	381	77.4 (41.2)	13.2 (9.0)	6.7 (2.6)	62.4 (25.3)
Range		5–320	1.6–60	1.2–17.2	34–262
*H. pylori status*^b^					
*H. pylori*−	37	66.1 (31.4)	8.45 (5.6)	8.4 (2.5)	52.9 (1 0 1)
*H. pylori*+*CagA*−	102	70.6 (31.4)	9.9 (4.7)	7.6 (2.6)	61.2 (23.6)
*H. pylori*+*CagA*+	229	80.9 (42.9)	15.5 (10.1)	5.9 (2.3)	64.7 (27.8)
*P*^c^		0.02	0.001	<0.0001	0.001
*Histological diagnosis^d^*					
Normal	42	70.4 (33.8)	8.7 (5.2)	8.4 (1.9)	53.7 (12.9)
NACG^e^	180	80.3 (40.2)	12.5 (7.3)	7.1 (2.4)	60.9 (18.6)
Antral atrophy	28	77.9 (36.2)	14.5 (11.3)	6.5 (2.3)	55.0 (13.2)
Corpus atrophy	27	70.1 (36.8)	17.2 (11.0)	4.5 (2.1)	73.9 (29.3)
Multifocal atrophy	7	57.9 (43.0)	16.7 (9.7)	3.6 (2.0)	85.7 (47.6)
*P*^c^		0.28	0.0001	<0.0001	0.0002

a Standard deviation;

b 13 missing data on *H. pylori* status;

c *P* value of ANOVA for PGA and the PGA/PGC ratioor Kruskal–Wallis test for PGC and gastrin;

d 97 missing data on histological diagnosis;

e nonatrophic chronic gastritis. *Note*: the difference in denominator between Tables 1 and 3 is because of five missing dataon gastrin measurement for five patients.

). Interestingly, except for PGC (*P*=0.015), there were no significant differences in the mean values of PGA, PGA/PGC, and gastrin levels between *H. pylori*-negative, *H. pylori*-positive, and CagA-negative patients (*P*=0.45, 0.14, and 0.12, respectively). However, there was an increase in the PGA mean value between *H. pylori*-positive and CagA-negative patients and *H. pylori*-positive and CagA-positive patients, which was even more accentuated for PGC and resulted in a significant decrease in the PGA/PGC ratio (*P*=0.03, <10^−3^, and <10^−3^, respectively). The mean values of PGA, PGC, and gastrin increased with the severity of the atrophy, while they decreased for PGA/PGC (Table 1, Figure 3

**Figure 3:**
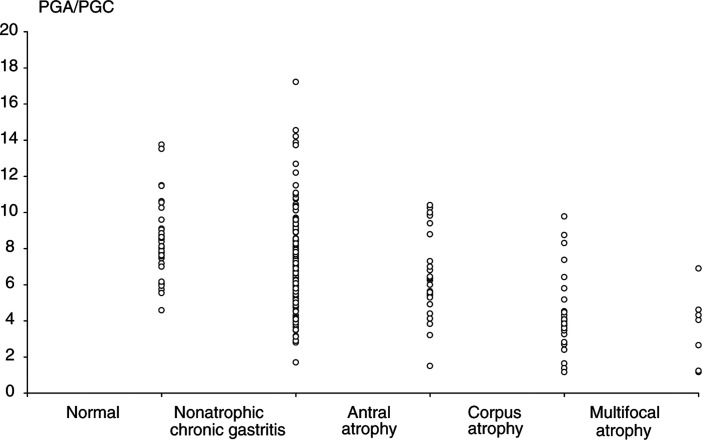
Distribution of the values of the PGA/PGC ratio according to histological diagnosis in dyspeptic patients. Results of the Eurohepygast study.

). The differences between mean values corresponding to each histological diagnosis were significant for PGA/PGC, PGC, and gastrin (*P*<10^−3^ for each of these biomarkers), but not for PGA (*P*=0.28) (Table 1). Interestingly, it was possible to differentiate three histological groups according to the values of their PGA/PGC mean: >7 for normal and inflammatory mucosa; <5 for corpus and multifocal atrophy; and an intermediate value between the previous two for antral atrophy (Table 1).

### Determination of the best threshold for each of the markers by means of the ROC curve

**Figure 4:**
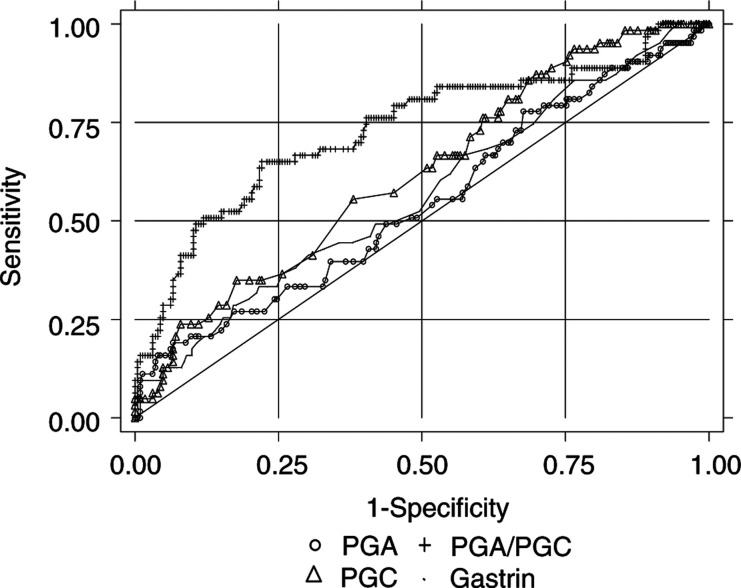
Unadjusted ROC curves of pepsinogen (PG) A, PGC, PGA/PGC, and gastrin to discriminate between patients with atrophic chronic gastritis (antral, corpus, or multifocal) and patients with normal or inflammatory gastric mucosa. Results of the Eurohepygast study.

The results of the ROC curves to discriminate between patients with ACG and patients with normal mucosa or non-ACG are based on the sample of 284 patients for whom histological diagnoses were available (Figure 4

). The areas under the ROC curves determined for PGA, PGC, PGA/PGC, and gastrin were 0.55, 0.62, 0.73, and 0.58, respectively, indicating that the PGA/PGC ratio was the only potential candidate as a useful biomarker for screening ACG in this population. The best PGA/PGC cut-off point for predicting ACG was 5.6. The corresponding validity parameters were: sensitivity, 65% (95% CI: 52.7, 76.1); specificity, 77.9% (95% CI: 72.1, 82.9); PPV, 45.1% (95% CI: 35.1, 55.3); and NPV, 88.9% (95% CI: 83.9, 92.7). The accuracy of the PGA/PGC ratio as a diagnostic test was 75%.

**Figure 5:**
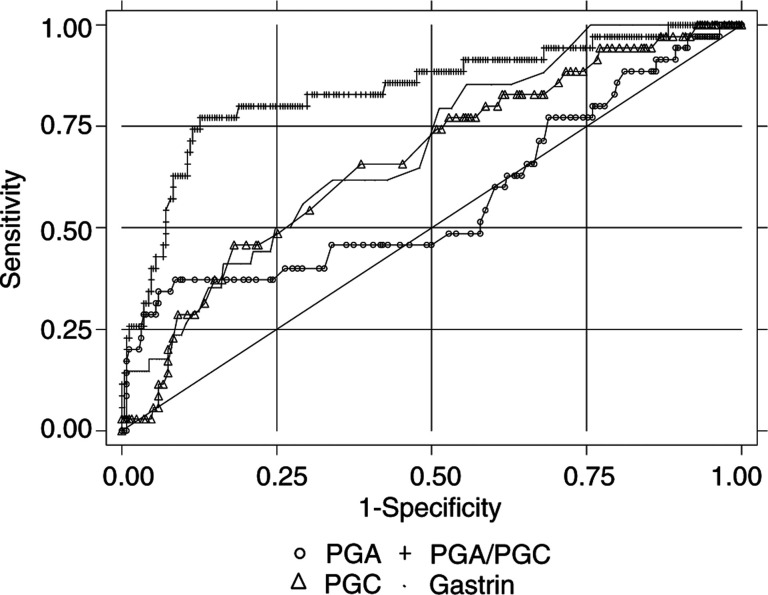
Unadjusted ROC curves of pepsinogen (PG) A, PGC, PGA/PGC, and gastrin to discriminate between patients with *H. pylori*-related corpus predominant or multifocal atrophy only and patients with normal, inflammatory, or antral atrophic gastric mucosa. Results of the Eurohepygast study.

The same procedure was applied to discriminate between patients with *H. pylori*-related corpus-predominant or multifocal atrophy and patients with normal, non-ACG, or antral atrophic mucosa. The areas under the ROC curves determined for PGA, PGC, PGA/PGC, and gastrin were 0.57, 0.67, 0.84, and 0.69, respectively (Figure 5

). Again, they indicated that the PGA/PGC ratio was the only potential biomarker for screening *H. pylori*-related corpus-predominant or multifocal atrophy in this population. The best PGA/PGC cut-off point for predicting *H. pylori*-related corpus-predominant or multifocal atrophy was 4.7. The corresponding validity parameters were: sensitivity, 77.1% (95% CI: 61.2, 88.8); specificity, 87.4% (95% CI: 82.9, 91.1); PPV, 45.8% (95% CI: 33.4, 58.5); and NPV, 96.5% (95% CI: 93.5, 98.4). The accuracy of the PGA/PGC ratio as a diagnostic test was 86.2%.

### Determination of independent variables explaining the variation of biomarkers by means of a multivariable linear regression

**Figure 6:**
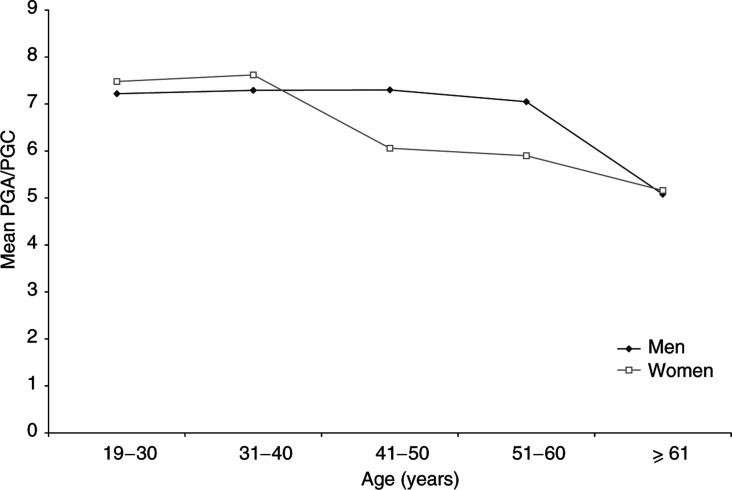
Mean PGA/PGC ratio according to age group and gender (interaction), in dyspeptic patients. Results of the Eurohepygast study.

A multivariable linear regression considering the PGA/PGC ratio as the outcome, was performed using all 34 variables available in the database. In the final linear model performed on the sample, few variables remained associated with a variation of PGA/PGC: presence of ACG, and presence of *H. pylori* and CagA antibodies. In this model, an interaction between age and gender was found, demonstrating a different age effect on the variation of PGA/PGC according to gender (Figure 6

). The PGA/PGC ratio decreased in women older than 41 years and in men older than 61 years. Accordingly, two linear regression models were constructed: one for women and one for men. In the model for women, the variable age was divided into two groups, younger than 41, and 41 years old or older; similarly, in the model for men, the variable age was divided into younger than 61, and 61 years old or older. In the model for women, in addition to the difference arising from age (coefficient=–1.35, *P*=0.001) for the older group compared to the younger group, *H. pylori*-positive and CagA-positive patients were more likely to have a lower PGA/PGC ratio (coefficient=–2.3, *P*=0.02) than *H. pylori*-positive CagA-negative patients (coefficient=−1.1, *P*=0.28) compared to *H. pylori*-negative patients (Table 2

**Table 2:** Multivariable linear regression of PGA/PGC, final model in men and in women. Results of the Eurohepygast study

	**Men**				**Women**			
	***n*=115**	**Coeff.^a^**	**95% Cl^b^**	***P***	***n*=162**	**Coeff.^a^**	**95% Cl^b^**	***P***
*Age*								
<61 years (men) or 41 years (women)	104	Baseline			63	Baseline		
⩾61 years (men) or 41 years (women)	11	−0.89	−2.16, −0.38	0.169	99	−1.35	−2.11, 0.60	0.001
*H. pylori and CagA status*								
*H. pylori*−	16	Baseline			13	Baseline		
*H. pylori*+CagA−	27	0.88	−0.72, 2.49	0.28	44	−1.11	−3.14, 0.92	0.28
*H. pylori*+CagA+	72	−1.07	−2.76, −0.62	<10^−3^	105	−2.29	−4.21, −0.37	0.020

a Coefficient adjusted on histological diagnosis;

b 95% confidence interval.

). In the model for men, no variables remained associated with the variation of PGA/PGC after adjustment by histological diagnoses. Concerning the results of PGA as an outcome in the multivariate linear model, only the variable linked to *H. pylori* and CagA status remained in the final model. The same results were observed for PGC. For gastrin, none of the variables evaluated remained significantly associated.

### Attempts to improve the performance of the PGA/PGC ratio for ACG

According to the results above, in order to discriminate between patients with ACG and patients with normal mucosa or non-ACG, a new cut-off point was determined by means of ROC curves drawn for each of the following three subgroups: men and women less than 41 years of age, and women 41 years of age or older. For each of the subgroups, the area under the ROC curve was 0.75, 0.76, and 0.68, respectively (Table 3

**Table 3:** Comparison of the performance of PGA/PGC ratio as a diagnostic test to detect histological lesions acccording to gender and age. Results of the Eurohepygast study

	**Diagnosis of ACG^a^**				**Diagnosis of corpus or multifocal atrophy**
	**Global sample**	**Men**	**Women<41 years old**	**Women ⩾ 41 years old**	**Global sample**
	***n*=289**	***n*=117**	***n*=70**	***n*=102**	***n*=289**
Area under ROC curve	0.73	0.75	0.76	0.68	0.84
Cut-off point	5.6	5.6	6.4	5.6	4.7
Sensitivity (%)	65.1	64	75	69.2	77.1
95% CI^b^	52.7, 76.1	44.1, 80.8	45.9, 93.2	49.8, 84.6	61.2, 88.8
Specificity (%)	77.9	84.8	70.7	65.8	87.4
95% CI^b^	72.1, 82.9	76.3, 91.1	58.1, 81.3	54.6, 75.8	82.9, 91.1
PPV^c^ (%)	45.1	53.3	34.6	40.9	45.8
95% CI^b^	35.1, 55.3	35.6, 70.5	18.4, 54.1	27.2, 55.8	33.4, 58.5
NPV^d^ (%)	88.9	89.6	93.2	86.2	96.5
95% CI^b^	83.9, 92.7	81.9, 94.8	82.6, 98.2	75.5, 93.4	93.5, 98.4
Accuracy (%)	75.1	80	71.4	66.7	86.2

a Atrophic chronic gastritis;

b 95% confidence interval;

c positive predictive value;

d negative predictive value.

). The cut-off point determined for each group was 5.6, 6.4, and 5.6, respectively. Dividing the sample into *H. pylori*-negative patients, *H. pylori*-positive and CagA-negative patients, and *H. pylori*-positive and CagA-positive patients did not improve the performance of the PGA/PGC ratio when compared to the performance in the total sample.

*H. pylori* and CagA status did not improve the performances of PGA and PGC after adjustment by the remaining variables from the multivariate linear model. This analysis was not carried out for *H. pylori*-related corpus-predominant or multifocal atrophy, because the subsamples were too small.

**Figure 7:**
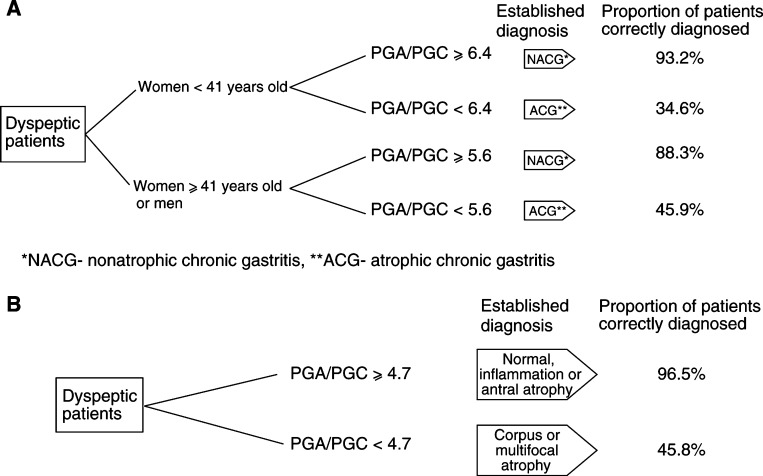
Proposed procedures and cut-off points of the PGA/PGC ratio to screen for gastric atrophy. Results of the Eurohepygast study: (**A**) to diagnose atrophic chronic gastritis (antral, corpus, or multifocal); (**B**) to diagnose *H. pylori*-related corpus-predominant or multifocal atrophy only.

From the present results, two independent procedures may be proposed, which require further evaluation (Figure 7

). The use of either procedure in a given population would depend on the type of atrophy that is being screened: ACG or *H. pylori*-related corpus/multifocal atrophy.

## DISCUSSION

The main finding of the present study was that the PGA/PGC ratio is an acceptable marker to screen for ACG, and a good marker for *H. pylori*-related corpus-predominant or multifocal atrophy. PGA, PGC, and gastrin alone were not appropriate as markers for either condition.

Interestingly, although PGC is secreted from different sites in the stomach, while PGA increases rapidly, PGC varies slightly, and Figure 2 indicates that PGC is a mathematical function of PGA. This relation may explain why the PGA/PGC ratio reflected stomach ACG more precisely than PGA alone. Therefore, although the PGA level theoretically reflects corpus atrophy; in this study, it was not a sufficiently good marker either for ACG or for *H. pylori*-related corpus-predominant or multifocal atrophy as already reported by Plebani *et al* (1996) in a study carried out in Italy. Also, Knight *et al* (1996) found that the PGA levels alone were not reliable markers for diagnosis of corpus atrophy in a population of male workers at high risk of gastric adenocarcinoma.

However, in the Eurogast study, Webb *et al* (1994) showed that the prevalence of low PGA levels was greater in countries with high incidence rates of gastric adenocarcinoma than in countries with lower rates.

The ability of PGA/PGC to discriminate between patients with ACG and patients with normal or inflammatory gastric mucosa was not as good as that found in studies which discriminated between severe atrophy of the corpus and normal mucosa alone (Borch *et al*, 1989). Indeed, the sample in the present study included patients with a wide range of histological manifestations. However, because of the differences in study design, in populations studied and in the definition of histological patterns, the comparison between the validity of the biomarkers has to be carried out cautiously.

A limitation of this analysis comes from the sample itself: (i) Because the sample is comprised of European patients consulting for dyspepsia in the Gastroenterology Department of teaching hospitals. (ii) Because all of these patients needed endoscopy, which selected patients with more severe symptoms. Therefore, it may not represent the global population of dyspeptic patients who may have a different prevalence of ACG. Indeed, it is impossible to obtain biopsies from a random sample of dyspeptic patients, that include those who are not referred to endoscopy as well as those who do not enter the health-care system. (iii) Histological diagnosis was available in 75% of these endoscoped patients, but there were no major differences between the original sample and the one analysed, thus allowing one to infer the results to the global sample of patients. Despite these limitations, three aspects of the methodology make this sample particularly valuable: (i) The best gold standard available, which is the review of slides by the same expert pathologist, was used. (ii) A large sample of endoscoped patients was included. (iii) The wealth of data available from the included patients allowed the analysis of many factors, which could influence the results of the biomarkers under study. Thus, these results can be applied without further validation to the consulting dyspeptic population referred to gastroscopy or high-risk populations for gastric cancer in Europe. However, the predictive values being dependent on the prevalence, if ACG prevalence was 10%, the NPV would increase to 95% and the PPV would decrease to 24.7%, and if ACG prevalence was 5% the NPV would be 97.7% and the PPV 13.4%.

Eventual differences in the prevalence of ACG among countries could result from an inclusion bias. For this reason, the country of origin was taken into account in the univariate analysis. On average, there was only a small number of ACG cases per country, and no difference was found among countries (data not shown), suggesting that the performances of the biomarkers in this sample and, in particular, their predictive values would not then vary among countries. However, the prevalence of ACG in the dyspeptic population in a given country would affect the PPV of the test used. The possible overestimation of *H. pylori* prevalence owing to misclassification of infected patients was certainly very small. Indeed, the three tests – serology, culture, and histology – were in good agreement: 122 of 156 cases (78.2%), including 104 positive and 18 negative.

To the best of our knowledge, the effects of serum storage duration under standard conditions on the accuracy of these hormone tests had never been formally evaluated. The mean storage time in this study was therefore compared between false positive/negative and true positive/negative samples and no differences were found. Furthermore, there were no major differences in the distribution of false-positive/-negative samples between the different centres, and the storage time was consistent between centres.

The decrease in the PGA/PGC ratio for women 41 years or older was not linked to an increase in the proportion of ACG in this age group. The proportion of ACG in each age group was not different for men and women (data not shown). Furthermore, after adjusting the two multivariate linear models (for men and women) by histological diagnosis, the relation between age and PGA/PGC disappeared in men, but not in women. It seems that in women aged 41 years or older, factors other than the presence of ACG may influence the variation of the PGA/PGC ratio. In the Eurogast study, Webb *et al* (1994) also found that the PGA level was lower in women and decreased with age. The role of female hormones may be worth investigating in this context. Among the other factors studied, the effect of CagA status in *H. pylori*-infected patients on the variation of PGA/PGC has been previously described (Kuipers *et al*, 1995; Webb *et al*, 1999).

In the context of epidemiological studies or clinical trials in which a large number of individuals or patients must be followed up or tested, a noninvasive, reliable test is of the utmost importance to improve the compliance of the subjects in the study, to minimise as much as possible the loss of data, to follow-up. The results presented here are an important starting point for (1) the validation in other independent samples of these threshold values determined for PGA/PGC, and the proposed procedures to discriminate among different histological entities; and (2) the analysis of the natural history of atrophy in these Eurohepygast patients for whom a 3-year follow-up has been carried out.

## Appendix 1

**The Eurohepygast Study Group:**

**Project leader:** Francis Mégraud (Université Bordeaux 2, France)

**Study coordinator:** Nathalie Broutet (Université Bordeaux 2, France)

**Steering Committee:** C O'Morain, President (Dublin, Ireland), P Sipponen (Espoo, Finland), A Price (London, UK), P Malfertheiner (Magdeburg, Germany), A Moran (Galway, Ireland), F Mégraud (Bordeaux, France), N Munoz (Lyon, France), F Dabis (Bordeaux, France), N Broutet (Bordeaux, France).

**Centralized Facilities:** N Figura (Siena, Italy), S Hynes (Galway, Ireland), A Moran (Galway, Ireland), M Plebani (Padova, Italy), A Price (London, UK), P Sipponen (Espoo, Finland), Torkel Wadström (Lund, Sweden).

**Coordinator of local centres:** IM Arenas (San Sebastian, Spain), A Blasi (Catania, Italy), JF Bretagne (Rennes, France), S Chaussade (Paris, France), JC Delchier (Créteil, France), JD De Korwin (Nancy, France), D Duda (Wroclaw, Poland), J Dzieniszewski (Warsaw, Poland), B Fixa (Hradec Kralové, Czech Republic), X Gisbert (Madrid, Spain), A Goldis (Timisoara, Roumania), J Guerre, (Paris, France), L Hryniewiecki (Poznan, Poland), G Inserra (Catania, Italy), AL Karvonen (Espoo, Finland), Z Knapik (Wroclaw, Poland), H Kolke (Tartu, Estonia), H Lamouliatte (Bordeaux, France), A Le Coustumier (Nancy, France), K Linke (Poznan, Poland), J Lonovics (Szeged, Hungary), H Maaroos (Tartu, Estonia), P Malfertheiner (Magdeburg, Germany), S Michopoulos (Athens, Greece), J Monés (Barcelona, Spain), C O'Morain (Dublin, Ireland), JG Pajarés, (Madrid, Spain), L Paradowski (Wroclaw, Poland), U Peitz (Magdeburg, Germany), S Pinto (Lisboa, Portugal), M Quina (Lisboa, Portugal), G Trautmann (Nancy, France), K Tchernev (Sofia, Bulgaria).
